# The role of maternal obesity in the risk of neuropsychiatric disorders

**DOI:** 10.3389/fnins.2015.00194

**Published:** 2015-06-18

**Authors:** Heidi M. Rivera, Kelly J. Christiansen, Elinor L. Sullivan

**Affiliations:** ^1^Division of Diabetes, Obesity, and Metabolism, Oregon National Primate Research CenterBeaverton, OR, USA; ^2^Department of Biology, University of PortlandPortland, OR, USA

**Keywords:** metabolic programming, attention deficit hyperactivity disorder, autism spectrum disorders, schizophrenia, mood disorders, eating disorders

## Abstract

Recent evidence indicates that perinatal exposure to maternal obesity, metabolic disease, including diabetes and hypertension, and unhealthy maternal diet has a long-term impact on offspring behavior and physiology. During the past three decades, the prevalence of both obesity and neuropsychiatric disorders has rapidly increased. Epidemiologic studies provide evidence that maternal obesity and metabolic complications increase the risk of attention deficit hyperactivity disorder (ADHD), autism spectrum disorders, anxiety, depression, schizophrenia, eating disorders (food addiction, anorexia nervosa, and bulimia nervosa), and impairments in cognition in offspring. Animal models of maternal high-fat diet (HFD) induced obesity also document persistent changes in offspring behavior and impairments in critical neural circuitry. Animals exposed to maternal obesity and HFD consumption display hyperactivity, impairments in social behavior, increased anxiety-like and depressive-like behaviors, substance addiction, food addiction, and diminished cognition. During development, these offspring are exposed to elevated levels of nutrients (fatty acids, glucose), hormones (leptin, insulin), and inflammatory factors (C-reactive protein, interleukin, and tumor necrosis factor). Such factors appear to permanently change neuroendocrine regulation and brain development in offspring. In addition, inflammation of the offspring brain during gestation impairs the development of neural pathways critical in the regulation of behavior, such as serotoninergic, dopaminergic, and melanocortinergic systems. Dysregulation of these circuits increases the risk of mental health disorders. Given the high rates of obesity in most developed nations, it is critical that the mechanisms by which maternal obesity programs offspring behavior are thoroughly characterized. Such knowledge will be critical in the development of preventative strategies and therapeutic interventions.

## Introduction

A third of women in the United States are obese (Ogden et al., 2012). Obesity not only leads to negative health consequences for the woman, but also for her child. The perinatal environment plays a crucial role in programming the normal development of a child's metabolic and mental health. Exposure to maternal obesity, excessive gestational weight gain (GWG), maternal metabolic disorders (diabetes, hypertension, and pre-eclampsia), and an unhealthy maternal diet adversely impact the behavior and physiology of children (Brekke et al., 2007; Olson et al., 2009; Stice et al., 2011; Maftei et al., 2015). Considering the concomitant rise in the prevalence of obesity and neuropsychiatric disorders (Boyle et al., 2011; Olfson et al., 2014), it is critical to understand the relationship between the two. Importantly, the increase in neuropsychiatric and neurodevelopmental disorders is likely due not only to maternal obesity, but also factors such as advancement in diagnostic tools and increased awareness. It should also be noted that obesity, excessive weight gain, and metabolic disorders during pregnancy are likely mediated not only through direct action on energy balance, but also through indirect effects of stress and mental health on food intake and physical activity. Epidemiological studies revealed that maternal obesity is a risk factor in the development of mental health disorders. However, due to constraints with studies involving humans, they failed to provide a causative link. Little is known about the role of excessive GWG, maternal metabolic disorders, and an unhealthy maternal diet on the development of mental health disorders. Animal models of maternal obesity, produced via high-fat diet (HFD) consumption, showed increased risk of behaviors associated with mental health disorders in offspring. Biological mechanisms potentially mediating these behavioral disorders include elevated systemic levels of nutrients (fatty acids, glucose), hormones (leptin, insulin), and inflammatory markers (C-reactive protein, interleukin, and tumor necrosis factor). These factors pass through the placenta into fetal circulation, producing permanent changes in offspring neuroendocrine regulation and neural development. In the developing offspring brain, inflammation impairs the establishment of neural circuits critical in regulating behavior, such as the serotoninergic (5-HT), dopaminergic (DA), and melanocortinergic systems. Impairments of these circuits increase the risk for developing mental health disorders.

This review has two main goals. First, we summarize studies that provide evidence for maternal obesity as a major risk factor for the development of mental health disorders in offspring. Each section highlights a different mental health disorder, presenting the relevant human and animal literature. We focus on the role of maternal obesity, excessive GWG, maternal metabolic disorders, and an unhealthy maternal diet on the development of mental health disorders in offspring, with a special emphasis on neurodevelopmental and neuropsychiatric disorders (Table 1). These disorders include attention deficit hyperactivity disorder (ADHD), autism spectrum disorder (ASD), anxiety, depression, schizophrenia, substance addiction, eating disorders (such as food addiction, anorexia nervosa, and bulimia nervosa), and cognitive impairments. Animal literature examines the role of maternal obesity (produced via HFD consumption) and impairments in offspring behavior, utilizing various models and behavioral assays (Table 2). Second, we discuss potential mechanisms by which maternal obesity programs behavior by impairing the development of neural circuitry, including 5-HT, DA, and melanocortin. The identification of risk factors that program mental health disorders in offspring, originating from impairments in the mother's metabolic intrauterine environment, will aid in the development of preventative strategies and therapeutic, dietary interventions.

**Table 1:** **Summary of human research demonstrating that maternal obesity increases the risk for mental health disorders**.

**Child outcome**	**Maternal factor**	**References**	**Study design**
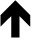 ADHD symptomology/Risk	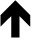 Pre-pregnancy BMI	Rodriguez et al., 2008	Cohort
		Rodriguez, 2010	Cohort
		Chen et al., 2014	Cohort
		Buss et al., 2012	Cohort
	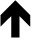 GWG	Rodriguez et al., 2008	Cohort
	Gestational diabetes & SES	Nomura et al., 2012	Cohort
		Schmitt and Romanos, 2012	Survey; Cohort
	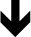 Dietary intake of omega-3 fatty acids	Field, 2014	Case-control
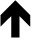 ASD risk/Severity of symptoms	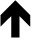 Pre-pregnancy BMI	Krakowiak et al., 2012	Case-control
		Reynolds et al., 2014	Cohort
		Moss and Chugani, 2014	Cohort
		Dodds et al., 2011	Cohort
		Bilder et al., 2013	Case-control; Cohort
	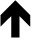 GWG	Dodds et al., 2011	Cohort
		Bilder et al., 2013	Case-control; Cohort
	Diabetes, hypertension, or pre-eclampsia	Krakowiak et al., 2012	Case-control
		Dodds et al., 2011	Cohort
		Lyall et al., 2012	Cohort
		Wallace et al., 2008	Cohort
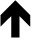 Anxiety/depression risk	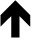 Pre-pregnancy BMI	Rodriguez, 2010	Cohort
		Van Lieshout et al., 2013	Cohort
		Colman et al., 2012	Cohort
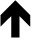 Schizophrenia risk	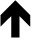 Pre-pregnancy BMI	Jones et al., 1998	Cohort
		Schaefer et al., 2000	Cohort
	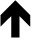 GWG	Kawai et al., 2004	Case-control
	Pre-eclampsia/hypertension and diuretic treatment	Dalman et al., 1999	Cohort
		Eide et al., 2013	Cohort
		Sorensen et al., 2003	Cohort
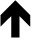 Food addiction	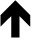 BMI 5 months post-delivery	Rising and Lifshitz, 2005	Cohort
	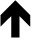 Intake of sweets during pregnancy	Brekke et al., 2007	Cohort
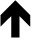 Anorexia nervosa/Bulimia nervosa risk	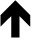 BMI 6 months post-delivery Disordered eating	Stice et al., 1999	Cohort
	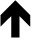 Intake of sweets during pregnancy	Lamerz et al., 2005	Survey
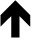 Risk of cognitive impairments	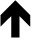 Pre-pregnancy BMI	Hinkle et al., 2012	Cohort
		Tanda et al., 2013	Survey; Cohort
		Neggers et al., 2003	Survey
		Heikura et al., 2008	Cohort
		Brion et al., 2011	Cohort
		Craig et al., 2013	Case-control

*Abbreviations: ADHD, attention deficit hyperactivity disorder; ASD, autism spectrum disorder; BMI, body mass index; GWG, gestational weight gain; SES, socioeconomic status*.

**Table 2:** **Summary of animal research demonstrating that maternal obesity leads to impairments in offspring behavior**.

**Offspring behavior**	**Maternal factor**	**Reference *Species***	**Experiment**
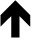 Hyperactivity	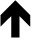 HFD consumption	Kang et al., 2014, *mouse*	Open field test
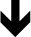 Sociability	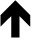 HFD consumption	Kang et al., 2014, *mouse*	3-chamber social interaction test
Attenuated sociability impairments	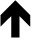 Choline consumption	Langley et al., 2014, *mouse*	3-chamber social interaction test
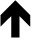 Anxiety-like behavior	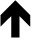 HFD consumption	Kang et al., 2014, *mouse*	Open field test
		Sullivan et al., 2010, *nonhuman primate*	Novel object test
		Bilbo and Tsang, 2010, *rat*	Morris water maze; elevated plus maze
		Sasaki et al., 2013, *rat*	Elevated plus maze; Open field test
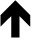 Depressive-like behavior	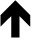 HFD consumption	Can et al., 2012, *rat*	Porsolt swim test
		Giriko et al., 2013, *rat*	Porsolt swim test
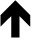 Substance addiction	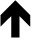 HFD consumption	Bocarsly et al., 2012, *rat*	Intermittent ethanol access test
		Morganstern et al., 2013, *rat*	Operant response to nicotine
		Naef et al., 2008, *rat*	Locomotor response to nicotine
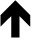 Food addiction	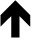 HFD consumption	Bayol et al., 2007, *rat*	Intake test
		Vucetic et al., 2010, *mouse*	Food preference test
		Naef et al., 2011, *rat*	Operant response to fat
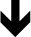 Cognition	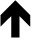 HFD consumption	Tozuka et al., 2010, *mouse*	Barnes maze

*Abbreviations: HFD, High-fat diet consumption*.

## Evidence from human and animal studies indicates that maternal obesity impacts offspring's risk for neurodevelopmental and neuropsychiatric disorders

### Maternal obesity as a risk factor for ADHD

ADHD is the most common neurodevelopmental disorder in the United States and is typically diagnosed during childhood or adolescence. ADHD is characterized by hyperactivity, cognitive impairments (problems with memory and focus), and impulsivity. According to the Center for Disease Control and Prevention, approximately 11% of children were diagnosed with ADHD in 2011 (Visser et al., 2014). The most recent estimate indicates that the prevalence of ADHD has risen by 33% (Visser et al., 2014) from 2003 to 2011. There is a substantial gender bias in ADHD with boys being twice as likely than girls to be diagnosed (Visser et al., 2014). Children with ADHD experience complications including poor academic performance, difficulties in peer relationships, substance abuse, unintentional injuries, and increased risk for delinquency (Hurley and Eme, 2004; Loe and Feldman, 2007). The increasing rate of ADHD and its comorbidity with other behavioral disorders present major challenges to the affected families and the nation's education and health care systems. Here, we discuss the evidence for maternal obesity, excessive GWG, maternal metabolic disorders, and an unhealthy maternal diet as potential risk factors for ADHD.

Research from three human studies indicates a link between maternal obesity and ADHD in children (Rodriguez et al., 2008; Rodriguez, 2010; Chen et al., 2014). Obese mothers had a two-fold increased risk of having a child with ADHD than their non-obese counterparts (Rodriguez et al., 2008). In addition, children of obese and overweight mothers displayed an increase in the severity of teacher-rated ADHD symptoms (inattention and difficulty dealing with emotions) than those of normal-weight mothers (Rodriguez, 2010). More recently, a Swedish study replicated these findings and demonstrated that obese and overweight mothers had children with an increased risk of ADHD (Chen et al., 2014). This association remained after adjusting for demographics (offspring sex, birth order, and maternal age). However, when familial factors, such as shared environment and genetics were controlled for this association was lost. Future work is needed to fully evaluate the role of familial factors in the link between maternal obesity and offspring ADHD risk. Animal research provides support that maternal obesity increases the risk of behaviors associated with ADHD. Mouse models of obesity, induced by HFD consumption, demonstrated that female offspring display decreased sociability (a key characteristic of ADHD) in a 3-chamber social interaction test, while male offspring display hyperactivity in an open field test (Kang et al., 2014). In summary, these studies provide evidence that maternal obesity is a risk factor for the development of ADHD and increased severity of ADHD symptomology in children. Studies that are able to elucidate the role of genetics and shared environment in this association are necessary. Moreover, the mechanisms by which maternal obesity increases a child's ADHD risk, such as epigenetic changes, need to be examined.

Excessive GWG and maternal metabolic disorders are also thought to impact the child's risk of ADHD. Two studies have examined the role of excessive GWG and risk for ADHD in children. A 2008 study found that mothers who were overweight and gained a large amount of weight during pregnancy showed a two-fold increased risk of their children displaying ADHD symptoms, such as hyperactivity and inattention (Rodriguez et al., 2008). However, another study found this association was lost after adjusting for impaired cognitive function in children (Buss et al., 2012), suggesting that excessive GWG also impacts offspring cognitive development and that deficits in cognition may partially mediate the GWG-ADHD link. Maternal metabolic disorders are also postulated to be associated with ADHD risk in children. Two studies have examined the impact of gestational diabetes mellitus on the development of ADHD in children (Nomura et al., 2012; Schmitt and Romanos, 2012). Both studies found that children of diabetic mothers had higher rates of ADHD symptoms. Offspring exposed to gestational diabetes mellitus had impairments in inattention but not in hyperactivity/impulsivity scores (Nomura et al., 2012). In families with low socioeconomic class, gestational diabetes increased offspring's risk for ADHD by 14-fold. However, this study had a relatively small sample size and relied on self-reported gestational diabetes diagnosis thus should be interpreted cautiously (Nomura et al., 2012). A second study with a larger sample size was able to confirm these findings (Schmitt and Romanos, 2012). The limited and conflicting findings of studies examining excessive GWG, maternal metabolic disorders, and risk for ADHD demonstrate the need for future studies that better track weight gain and metabolic disorders during pregnancy.

There is limited evidence on the impact of an unhealthy, maternal diet on offspring ADHD risk. However, recent evidence indicates that a maternal diet deficient in omega-3 fatty acids increases risk for ADHD. This case control study identified deficient omega-3-fatty acid levels as a nutritional risk factor for ADHD (Field, 2014). Multiple causes for this deficiency were proposed to originate from either variation in the amount of fatty acid metabolism genes, differences in the level of fatty acid intake necessary for normal development between sexes, impaired placental transport of long-chain polyunsaturated fatty acids, particularly docosahexaenoic acid and arachidonic acid, which are critical for the development of the fetal central nervous system. This deficiency in omega-3-fatty acids may be mediated through impairment of materno-fetal long chain polyunsaturated fatty acid transport in women with gestational diabetes (Larqué et al., 2011). Omega-3-fatty acid supplementation may represent a promising dietary intervention. However, the degree to which gestational diabetes affects fatty acid transfer from the mother to the developing child needs to be clarified.

### Maternal obesity as a risk factor for ASD

ASD encompasses a wide spectrum of developmental disorders characterized by impaired social skills and includes restricted or repetitive interests and activities. The cognitive abilities of those with ASD can vary greatly from extremely high functioning and gifted, to severely challenged. The prevalence of ASD has increased 30% over the last decade (Wingate et al., 2014). As with ADHD, there is a gender bias in ASD diagnosis, with males being 5 times more likely than females to be diagnosed. ASD not only places a severe emotional strain on families, but is an economic burden as well. Importantly, early diagnoses and interventions are critical as they improve long-term outcomes and reduce lifetime costs to the patient and family (Horlin et al., 2014). Here, we will discuss the evidence for perinatal metabolic and nutritional risk factors for ASD.

Three human studies provide evidence of a link between maternal obesity and ASD in children (Krakowiak et al., 2012; Moss and Chugani, 2014; Reynolds et al., 2014). Krakowiak and colleagues were the first to demonstrate that maternal obesity increased risk of ASD diagnosis and developmental delay in children (Krakowiak et al., 2012). This finding was supported by a study reporting that maternal obesity increased the risk of ASD diagnosis in toddlers (Reynolds et al., 2014). Maternal obesity was also linked to a delay in language skills in this study. Other studies have demonstrated an indirect relationship where maternal obesity increased the likelihood of low birth weight, which was associated with a two-fold increased risk of ASD diagnosis (Moss and Chugani, 2014). In a large-scale population based study, Dodds et al. observed that maternal pre-pregnancy weight greater than 90 kg was a risk factor for offspring developing ASD (Dodds et al., 2011). These findings were not in agreement with a study by Bilder and colleagues, where excessive GWG, not pre-pregnancy BMI, increased risk for ASD in children (Bilder et al., 2013), indicating interplay between GWG and maternal obesity on a child's ASD risk. The majority of studies demonstrate that maternal obesity is a risk factor for ASD diagnosis and developmental delay in children. However, these studies are largely underpowered, and several occurred in non-US populations in the 1970s and 1990s, with obesity being much less prevalent. Also, most of these studies suffered from methodological limitations including large attrition (Hinkle et al., 2013; Moss and Chugani, 2014), sampling biases for control groups (Stein et al., 2006; Krakowiak et al., 2012), reliance on parental report to evaluate past exposure and current offspring diagnostic status (Stein et al., 2006; Hinkle et al., 2012, 2013; Moss and Chugani, 2014), lack of statistical power (Stein et al., 2006), and inability to adjust for confounders (Stein et al., 2006; Dodds et al., 2011). Despite these shortcomings, the results point to an aversive impact of maternal obesity on developmental outcomes, including ASD and other delays.

The role of excessive GWG on ASD risk in children has only been examined by two studies. Dodds et al. showed that GWG greater than 18 kg presented an increased risk for ASD in children (Dodds et al., 2011). The analysis of both cohorts in the study conducted by Bilder et al. demonstrated that GWG of over 11 kg was associated with risk of ASD in children (Bilder et al., 2013). Four studies have examined the relationship between maternal metabolic disorders and the risk for ASD in children. Krakowiak et al. found positive associations with gestational diabetes, hypertension, and pre-eclampsia and ASD risk (Krakowiak et al., 2012). These metabolic disorders were associated with an even greater risk of developmental delays (Krakowiak et al., 2012). Diabetes, in particular, was associated with verbal developmental delay. In a large cohort study, maternal complications, including gestational diabetes and hypertension, were associated with an increased risk of ASD in children (Lyall et al., 2012). Wallace et al. showed that gestational hypertension and pre-eclampsia, but not gestational diabetes, were associated with increased severity of ASD symptoms (Wallace et al., 2008). Dodds et al. showed that gestational diabetes and hypertension were positively correlated with ASD diagnosis (Dodds et al., 2011). The findings of the few existing studies demonstrate the need for future studies to better track weight gain during pregnancy and the interaction between pre-pregnancy body weight and optimal GWG need to be examined. Also, the limited evidence that exists indicates that diabetes and hypertension during pregnancy may increase the risk for ASD and developmental delays in children.

To date, two human studies have directly examined the fatty acid composition of the mother's diet on the child's ASD risk. The first study found that intake of high levels of omega-6 fatty acids, such as linoleic acid, reduced the risk of having a child with ASD by 34% (Lyall et al., 2013). The second case control study identified deficient omega-3-fatty acid levels as a nutritional risk factor for offspring ASD (Field, 2014). Interactions between pre-existing maternal psychopathology and breastfeeding were also identified as potential risk factors for ASD (Field, 2014). In rodent models, maternal HFD consumption resulted in deficits in social behavior in female offspring (Kang et al., 2014) and choline supplementation during pregnancy alleviated deficits in offspring social interaction (Langley et al., 2014). Further studies investigating the impact of maternal diet on offspring ASD risk are warranted. Moreover, the protective effects of supplementation with omega-3 and omega-6 fatty acids need to be investigated. If initial findings are supportive, omega-3 and 6 fatty acid supplementation could be encouraged as a cost-effective prophylactic for ASD.

### Maternal obesity as a risk factor for anxiety and depression

Anxiety and depression are among the most common neuropsychiatric disorders in children and adolescents in the United States. Anxiety is diagnosed as persistent worry or fear that leads to impairment of daily life, whereas depression is characterized by dysphoria. The prevalence of anxiety and depression is 8% in children and adolescents and continues to rise (Eapen and Crncec, 2012). Complications associated with anxiety and depression, include poor social interactions, strained familial relationships, and negative self-worth (Dweck and Wortman, 1982; Strauss et al., 1989; Bernstein et al., 1996; Masi et al., 1999). Here, we will discuss the evidence for perinatal metabolic and nutritional risk factors for anxiety and depression.

Research has demonstrated a link between maternal obesity and the development of anxiety and depression in children and adolescents, which have been examined in a total of three studies. Maternal pre-pregnancy BMI increased the risk of disrupted emotions (e.g., fear and sadness) and has also been associated with increased internalizing behaviors (behaviors associated with withdrawal and depression) in children (Rodriguez, 2010; Van Lieshout et al., 2013). These findings suggest that maternal obesity is involved in the disruption of emotions. In support of this finding, but in a more indirect manner, another group demonstrated that maternal obesity increases the likelihood of low or high birth weight (Nohr et al., 2008; Djelantik et al., 2012; Gavard and Artal, 2014), which is also correlated with development of anxiety and depression in adolescents (Colman et al., 2012). Additionally, in adults, a gender bias has been observed in these disorders with the association between obesity and anxiety being stronger in women than men (Desai et al., 2009) suggestive of an interaction between sex and metabolic hormones. More research is needed to examine whether a gender bias is also observed in a younger population. However, it has proven to be difficult to diagnose children. These children are still developing, thus symptoms may go unnoticed as they are attributed to developmental changes. Furthermore, children with anxiety and depression do not express any physical symptoms further impairing diagnosis.

Rodent research provides further support that maternal obesity (induced via HFD consumption) increases anxiety-like behavior in offspring; however, it disagrees as to the sex affected. Presently, three major rodent studies exist. The Morris water maze, commonly used to assess anxiety-like behavior, exploits a rodents' natural desire to escape from water. Swim speed indicates how motivated an animal is to escape, a measure of stress level. Male HFD offspring displayed more anxiety-like behavior than females in this test (Bilbo and Tsang, 2010). They swam faster in the Morris water maze, indicating increased motivation to escape. In the elevated plus maze, rodents have an innate tendency to hide from predators by spending most of their time in the dark and avoiding bright open spaces. Male HFD offspring spent less time in the open arms of an elevated plus maze relative to controls, indicating increased anxiety. Female offspring displayed increased anxiety-like behavior, regardless of maternal diet, in the Morris water maze and elevated plus maze. Sex differences can be attributed to the behavioral test administered. In one study, female offspring made fewer entries into the open arms of an elevated plus maze, whereas male offspring spent less time in the center of an open field test (Sasaki et al., 2013). In another study, female HFD offspring displayed anxiety-like behavior, as they made less visits to the center of an open field test, whereas the males were hyperactive (Kang et al., 2014). Additionally, two rodent studies have indicated that maternal HFD increases depressive-like behavior, as offspring spent less time swimming and climbing in the Porsolt swim test (indicating less escape attempts) (Can et al., 2012; Giriko et al., 2013). However, these studies only examined male offspring. Future rodent studies need to use the same maternal diet composition/duration, behavioral assay, sex and age of offspring to be able to compare across studies. Despite inconsistencies in sex, the benefit of using rodent models is they are cost-effective, yield a large sample size, and provide insight into the mechanism involved.

Our laboratory has developed a nonhuman primate (NHP) model of diet-induced obesity. NHPs are an important model as they develop the full spectrum of metabolic disease, express complex social behaviors, and have similar development of the brain as humans. A major strength of our model is that we can assess the impact of maternal diet and metabolic status, independently, on the development of mental health disorders (McCurdy et al., 2009). We found that maternal HFD consumption and obesity increases anxiety-like behavior in female offspring, as demonstrated by a greater latency to touch a potentially threating novel object (Sullivan et al., 2010). Male HFD offspring displayed increased aggression during the human intruder test used to assess temperament. There was a gender bias with females being more prone to anxiety-like behavior and males being more prone to aggression, which is consistent with evidence found in humans (Archer, 2006; Desai et al., 2009). These studies provide initial evidence that maternal diet and obesity is involved in the development of anxiety and depression in offspring, specifically with respect to difficulty regulating emotions. The role of additional maternal metabolic impairments on the development of anxiety and depression remain unstudied.

### Maternal metabolic state and risk for schizophrenia

Schizophrenia is a disabling disorder characterized by hallucinations or delusions, disorganized speech, flat affect, and impairments in cognition. The prevalence of schizophrenia is on the rise (Stevens et al., 2014). The majority of studies demonstrating that maternal metabolic state impacts offspring risk of developing schizophrenia originate from historical famine events. Developmental exposure to malnutrition was first linked to schizophrenia risk in a classic study conducted on individuals who suffered from the Dutch Hunger Winter in 1944–1945 (Stein et al., 1975). In Western countries, obesity, instead of famine is believed to impact risk of schizophrenia. Here, we discuss the evidence for perinatal metabolic factors for schizophrenia.

Six human studies have indicated a link between maternal obesity and schizophrenia. A case control study found a 24% increase in schizophrenia risk for every BMI unit increase during early pregnancy and 19% during late pregnancy (Kawai et al., 2004). Two cohort studies reported a two-fold increased risk for schizophrenia in offspring with an elevated maternal BMI (Jones et al., 1998; Schaefer et al., 2000). Schaefer et al. examined a cohort of approximately 19,000 births from 1959 to 1967 in Alameda County, California. Their findings showed a three-fold increased risk of schizophrenia with an elevated maternal BMI (Schaefer et al., 2000). The other study focused on a Finnish cohort of approximately 11,000 participants either born on or after 1966 (Jones et al., 1998). Their findings showed a two-fold increased risk of schizophrenia with an elevated maternal BMI, though this association was no longer evident after gender, social class, and age of mother at conception were controlled for (Jones et al., 1998). It is especially important that maternal age is controlled for in these studies as advanced maternal age is associated with increased maternal body weight and increased risk of offspring developing neurodevelopmental disorders. These studies provide preliminary evidence that maternal obesity may be a risk factor for offspring developing schizophrenia. Additional cohort studies that examine more ethnically diverse study populations and control for important socio-demographic factors are needed to determine the broad translatability of these findings.

The role of maternal metabolic disorders (gestational diabetes, hypertension, and pre-eclampsia) and offspring risk of schizophrenia has been examined in four studies. A positive association between maternal pre-eclampsia and risk for offspring schizophrenia was reported in two studies (Dalman et al., 1999; Eide et al., 2013); with one study reporting a 2.5-fold risk (Dalman et al., 1999). A positive association between maternal hypertension and schizophrenia was demonstrated in a third study (Sorensen et al., 2003). The risk of schizophrenia increased four-fold in offspring of mothers with hypertension treated with diuretics (Sorensen et al., 2003). Importantly, it is unclear if the higher risk is due to the increased severity of hypertension that required diuretic treatment or to gestational exposure to the diuretic itself. Lastly, preliminary evidence indicated that mothers of offspring with schizophrenia were seven times more likely to have diabetes during pregnancy. However, due to the small sample size and high variability between groups this was not statistically significant (Jones et al., 1998). Despite, a few studies alluding to an association between altered metabolic states, like diabetes and pre-eclampisa, and offspring's risk of schizophrenia, additional research is needed to fully understand mechanisms contributing to schizophrenia. Furthermore, no animal research has been conducted. These studies provide preliminary evidence for an association between maternal obesity and schizophrenia. Limited evidence also indicates that maternal metabolic disorders may be a risk factor for developing schizophrenia.

### Maternal obesity as a risk factor for substance abuse and addiction

In the United States, substance abuse is a major parental concern. Often beginning early in life, substance abuse is characterized by a maladaptive pattern of substance use that causes distress. Chronic substance abuse can lead to addiction that manifests as the loss of control over drug intake (binge), negative symptoms if drug is removed (withdrawal), and constant worry that impairs daily life (Koob et al., 2008). Notably, two out of three high school students have consumed alcohol (Johnston et al., 2015). Further, the prevalence of substance abuse is 8% in adolescents (Rowe, 2012). This high rate of substance abuse leads to medical complications ranging from acquisition of sexually transmitted diseases up to death caused by overdose or accidents (Schulte and Hser, 2014). Here, we discuss the evidence for maternal obesity as a risk factor for substance addiction in offspring. The lack of human studies forces us to rely on the animal literature.

No single animal model can fully represent human addiction, rather each experimental animal model mimics a different aspect of the disease, e.g., acquisition phase (recreational substance use), escalation phase (substance abuse), extinction phase (abstinence), and reinstatement phase (relapse) (Robinson, 2004). Using rodent models, three studies have examined the link between maternal obesity and risk for substance addiction in offspring. One group utilized an intermittent ethanol-access schedule adapted from food addiction studies (12 h ethanol access per day) to induce an ethanol binge (Bocarsly et al., 2012). Adult offspring from mothers that consumed a HFD during gestation exhibited an increase in ethanol intake relative to control offspring, indicating increased susceptibility to ethanol binging. Employing assays more commonly used in substance addiction, one group examined the influence of maternal HFD consumption on operant responses to nicotine (Morganstern et al., 2013). Maternal HFD offspring displayed enhanced operant responses to nicotine during acquisition (using fixed-ratio and progressive-ratio schedules of reinforcement) and escalation demonstrating increases in the rewarding properties of nicotine. An unexpected diminished response was found during reinstatement. This diminished response may be attributed to the development of tolerance after chronic substance use due to downregulation of nicotinic receptors. This hypothesis is supported by previous research that maternal HFD offspring had a diminished locomotor response to chronic treatment with the stimulant amphetamine (Naef et al., 2008). However, this story is not straightforward as maternal HFD offspring also have a diminished locomotor response to acute amphetamine treatment. The animal literature provides evidence that an unhealthy maternal diet impairs the rewarding properties of substances of abuse in offspring. Further research is needed to examine why maternal HFD selectively targets addiction phases, the directionality of the effect, and the underlying mechanisms involved. The paucity of human studies on substance abuse and addiction should not be interpreted as a lack of effect. Additional clinical research is needed to better characterize the effect of maternal obesity, excessive GWG, maternal metabolic disorders, and an unhealthy maternal diet on the development of substance abuse and addiction. This will help assess the translational utility of animal models involving substance addiction.

### Maternal obesity as risk factor for food addiction

The number of papers published on food addiction has risen dramatically from 53 articles in 2003 to 279 in 2013, a five-fold increase. The growing interest in food addiction has been sparked by ongoing debate as to whether or not it should be classified as an addiction (Avena et al., 2012; Ziauddeen et al., 2012). Consequently, only two studies exist in the human literature that have examined the link between maternal obesity and food addiction in children and infants. Using a laboratory setting, one group found that infants of obese mothers had a drive to overeat food with a high carbohydrate content in comparison to infants from normal-weight mothers (Rising and Lifshitz, 2005). However, no differences were observed in the consumption of food with a high protein or fat content. Another group examined the influence of a mother's unhealthy diet during pregnancy on food intake, food choice, and meal patterns in children (Brekke et al., 2007). The drive to overeat sweets in children was positively correlated with the mother's overconsumption of sweet foods. Children were introduced to sweets earlier (25 days earlier) and were allowed to eat sweets more frequently (more than 1 or 2 times per week) than children with mothers that did not overeat sweets. These children also consumed less healthy food (fruit and vegetables). These studies provide evidence that maternal obesity and diet program a drive to overeat food in offspring. However, these studies used food frequency questionnaires that are prone to errors (Kristal et al., 2005). Additional human studies that examine maternal and offspring diet using 24-h recall to examine the impact of maternal diet on offspring food addiction are warranted. More studies are also needed to investigate the association between maternal obesity, maternal metabolic disorders, and excessive GWG on food addiction in offspring.

Evidence from three rodent studies provides further support that maternal obesity increases the risk of food addiction in offspring. An earlier study demonstrated that rat offspring whose dams consumed a “junk food” diet (ranging widely in content from potato chips to marshmallows), during gestation and lactation, and who themselves consumed a “junk food” diet after weaning, had a drive to overeat this same diet in comparison to control offspring (Bayol et al., 2007). However, the diversity of ingredients in this “junk food” diet prevents identification of the specific food(s) mediating this programming effect. A later study where dams were fed a more controlled-diet high in fat content, during gestation and lactation, found that offspring had a drive to overeat palatable food high in sugar and fat content (Vucetic et al., 2010). Moreover, one study examined whether maternal HFD alters the rewarding properties of food using a protocol typically used to study drug addiction. This study found that maternal HFD offspring displayed enhanced operant responses to fat (using fixed-ratio and progressive-ratio schedules of reinforcement), but not to sucrose (Naef et al., 2011). This is the first study to identify that an unhealthy maternal diet enhances the rewarding properties of food. Future studies will be needed to replicate this finding and to better characterize the addiction phases targeted by maternal HFD.

### Maternal obesity as risk factor for anorexia and bulimia nervosa

Children and adolescents may suffer from eating disorders, such as anorexia nervosa and bulimia nervosa. Anorexia nervosa is characterized by restriction of food intake, low body weight, and fear of gaining weight. Bulimia nervosa is characterized by recurring episodes of binge eating and compensatory behavior to reduce weight gain. A recent study conducted in Australia asserted that the prevalence of eating disorders is higher than previously believed (Allen et al., 2013) with a clear gender bias for females (females = 10–15%, males = 3–4%). Particularly troubling, eating disorders have the highest mortality rate of all neuropsychiatric disorders (Harris and Barraclough, 1998). We briefly discuss two studies that examine the influence of maternal obesity as a risk factor for eating disorders in children. In a preliminary study, one group demonstrated that maternal obesity (at 6 months post-delivery) predicted the onset of inhibited and secretive eating over the first 5 years of a child's life (Stice et al., 1999). This behavior indicated that the children experienced elevated worry concerning weight gain. In addition, this group reported that maternal eating patterns (bulimic symptoms, disinhibited eating, hunger, and body dissatisfaction) were associated with the onset of secretive eating in children. Therefore, the children could have modeled their eating patterns after their mothers. A cross-sectional study conducted in Germany also found that children whose mothers suffered from binge eating had a six to seven-fold increased risk of developing binge and night eating (Lamerz et al., 2005). These studies suggest that maternal obesity and eating patterns influence the onset and risk for disordered eating behavior in children. However, when these studies were conducted standardized tests were not yet established; thus neither study used standardized assessments to examine the children's eating patterns. Future studies should replicate these experiments using standardized questionnaires, such as the Eating Disorders Diagnostic Interview later developed by the same research group [Stice et al. (Rohde et al., 2015)], to improve reliability and validity. The influence of excessive GWG, maternal metabolic disorders, and unhealthy maternal diet on the development of eating disorders remains unknown. Considering, no animal research has been conducted on this topic, studies utilizing activity-based anorexia or binge-eating models should be performed in order to compare with human studies and elucidate the mechanisms that underlie this relationship.

### Maternal obesity as risk factor for cognitive impairments

Maternal obesity has been associated with cognitive impairments in offspring. Children of obese mothers were reported to have decreased cognitive and language scores (Hinkle et al., 2012). A recent study confirmed this finding by demonstrating that maternal obesity was associated with a reduction in child's reading and math scores on standardized tests after intrauterine, family background, maternal, and child factors were controlled for (Tanda et al., 2013). These findings were further supported by a study by Neggers et al. that demonstrated an association between maternal obesity and a reduction in offspring IQ in a low socioeconomic African American population (Neggers et al., 2003). Also, maternal obesity was linked to increased incidence of mild intellectual disability in a Finnish cohort (Heikura et al., 2008). However, a study by Brion and colleagues did not demonstrate an association between offspring cognitive test scores and maternal obesity (Brion et al., 2011). Another study found an association between maternal obesity and decreased language scores in 8 year old children with obese mothers, but no cognitive deficits (Craig et al., 2013). These inconsistencies between human studies likely reflect differences in demographic factors, obesity prevalence between study populations, and differences in the scales used and measures assessed. A more standardized scale would prove beneficial to assess these cognitive impairments and to compare across research groups. Animal studies provide support to the notion that exposure to maternal obesity impairs offspring cognition. Offspring of mothers that consumed a HFD were associated with delayed spatial learning using the Barnes maze in young rats (Tozuka et al., 2010). Additional human studies that examine large diverse study populations are needed to fully examine the impact of maternal obesity on offspring cognition. Also, further animal studies are warranted that elucidate the mechanisms by which maternal obesity may decrease offspring cognition.

### Possible mechanisms by which maternal obesity increases risk for mental health disorders

#### Inflammation-induced programming

**Figure 1:**
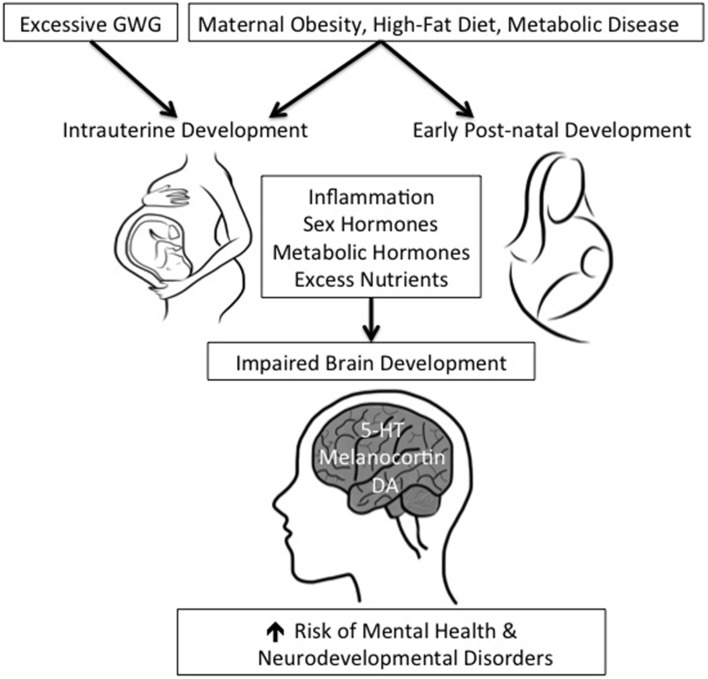
**Potential mechanisms through which maternal obesity, metabolic state, high-fat diet, and excessive gestational weight gain may lead to the development of mental health disorders in offspring**. Maternal obesity, metabolic disorders, and high-fat diet impact both intra-uterine and early post-natal development. Excessive gestational weight gain impacts intrauterine development. Maternal obesity produces systemic inflammation (via increased pro-inflammatory cytokines) and dysregulation of metabolic hormones. These cytokines/hormones cross over into the fetal circulation via the placenta. During development, enhanced inflammation of the brain is a major contributor to impairments in neural circuits critical for regulation of behaviors, such as 5-HT, DA, and melanocortin. A gender bias has also been observed in the development of mental health disorders, therefore, sex hormones may also play a role in impairing brain function; the location of its action is poorly described.

Obesity is considered a state of chronic inflammation as an increase in adipose tissue mass results in greater secretion of inflammatory markers including C-reactive protein, interleukin-6, interleukin-1β, and tumor necrosis factor-α (Das, 2001) (Figure 1). The inflammatory state associated with obesity is postulated to underlie many of the associated metabolic diseases including cardiovascular disease, heart disease, insulin resistance, diabetes, and hypertension (Das, 2001). Elevated inflammatory cytokines are also observed in obese pregnant women and lead to dysfunction in the endothelium (Stewart et al., 2007) and placenta (Leung and Bryant, 2000; Nordahl et al., 2008). Maternal HFD increases the amount of the inflammatory cytokine, IL-1β, in the NHP hypothalamus (Grayson et al., 2010). Increased inflammatory factors are associated with neurodevelopmental [ASD (Goines et al., 2011; Ashwood et al., 2011a,b) and ADHD (Oades et al., 2010; Oades, 2011)] and neuropsychiatric disorders [anxiety (Maes et al., 2002; Terness et al., 2002) and depression (Zanoli et al., 2001; Terness et al., 2002)]. Mounting evidence indicates that developmental exposure to elevated pro-inflammatory cytokines influences brain development. Specifically, circulating pro-inflammatory cytokine levels impact the development of neural circuits critical for the regulation of behavior (Das, 2001). Thus, inflammation is an important potential mechanism by which exposure to maternal obesity and HFD consumption impairs offspring brain development and increases risk for mental health disorders.

#### Sex hormone-induced programming

Some of the most dramatic findings in psychopathology are the marked gender differences in neurodevelopmental and mental health disorders. However, biological mechanisms mediating this gender bias have not been clearly defined. Sex hormones could impact brain function through altered hormones in the periphery, a direct action on receptors in the brain, or both. Gender differences in brain development are programmed by androgen production by the fetal testes during critical periods of development. In primates, the fetal testes begin producing androgens at the end of the first trimester, with production continuing into the second semester, and a surge at birth (Forest et al., 1973; Gendrel et al., 1980). Prenatal androgens regulate gene and protein levels of important transcription factors involved in development, such as forkhead box protein P2 (Bowers et al., 2014). Supporting a central effect, sex hormones are known to regulate inflammation in the brain (Pozzi et al., 2006) and signaling of neural circuits critical for regulating behavior (Bethea et al., 2002; Al Sweidi et al., 2012). Androgens also result in sex differences in immune cells including microglia (Lenz et al., 2013; Lenz and McCarthy, 2014) and mast cells (Lenz and McCarthy, 2014). Microglia are critical for brain development as they play an important role in proliferation, synaptogenesis, cell survival, programmed cell death, and synaptic pruning (Lenz and McCarthy, 2014). Sex differences are also observed in inflammatory cytokines and chemokines in the developing hippocampus, amygdala, and cortex (Schwarz et al., 2012). We hypothesize that sex differences in the response of the innate immune cells to maternal obesity-induced inflammation underlies the observed gender differences in behavior. As development of the brain circuitry critical in behavioral regulation are sensitive to inflammatory cytokines (Jarskog et al., 1997; Ishikawa et al., 2007), we believe that gender differences in behavior are due to unique inflammatory-induced changes in the development of this brain circuit (Spinelli et al., 2010).

#### Metabolic hormone-induced programming

Maternal obesity is associated with gestational diabetes, maternal hyperglycemia, hyperinsulinemia, and hyperleptinemia (Leung and Lao, 2000). A pregnancy complicated by maternal obesity and metabolic disorders exposes the developing offspring to increased circulating nutrients (fatty acids, glucose), and metabolic hormones (leptin, insulin). Glucose, but not insulin, can pass through the blood-placenta-barrier and enter the fetal circulation (Oken and Gillman, 2003). Consequently, the fetal pancreas compensates with increased insulin release. Maternal-fetal transmission of leptin has also been observed in the circulatory system (Luo et al., 2013). Leptin receptors are widely distributed in brain regions involved in behavioral regulation including the cortex, hippocampus, amygdala, thalamus, and hypothalamus (Couce et al., 1997; Meister, 2000). Leptin has also been shown to increase inflammatory cytokine secretion and may also indirectly influence brain development (Lappas et al., 2005). Elevated levels of metabolic hormones and nutrients during the perinatal period alter the development of the neural circuits critical for the regulation of energy balance and behavior (Harder et al., 1998; Proulx et al., 2002).

#### Maternal obesity impairs development of the serotonin (5-HT) system

A potential mechanism for the increased risk of mental health disorders associated with early exposure to maternal obesity is abnormal development of the 5-HT system. The 5-HT system is a key player in the regulation of emotion and dysregulation of the 5-HT system is linked to numerous mental health disorders. 5-HT plays a crucial role in neural development, influencing synapse formation, neurogenesis, and the migration of neurons (Daws and Gould, 2011; Kannan et al., 2011). We observed a decrease in 5-HT synthesis in NHP offspring exposed to maternal HFD-induced obesity resulting in increased anxiety-like behavior in female offspring (Sullivan et al., 2010). This finding is supported by a rodent study in which rodent HFD offspring displayed an increase in inhibitory autoreceptors (known to inhibit 5-HT release) (Peleg-Raibstein et al., 2012). A reduction in 5-HT production underlies neurodevelopmental disorders, such as ADHD (Oades et al., 2008) and ASD (Chugani et al., 1999; Challier et al., 2008), and neuropsychiatric disorders, including anxiety (Kiyohara and Yoshimasu, 2009) and depressive disorders (Sullivan et al., 2006; Laucht et al., 2009; Spindelegger et al., 2009). Moreover, selective serotonin reuptake inhibitors are a standard treatment for these disorders. Thus, several lines of evidence indicate that changes in the development of the 5-HT system are a plausible mechanism for maternal obesity increasing the risk of neurodevelopmental and neuropsychiatric disorders in offspring. We hypothesize that the increased production of inflammatory factors associated with maternal obesity suppresses the development of the 5-HT neural circuit. Treatment with pro-inflammatory cytokines, such as interferon-α, reduces the axonal density of 5-HT neurons in brain regions critical in behavioral regulation such as the prefrontal cortex and amygdala (Ishikawa et al., 2007). Also, pro-inflammatory cytokine treatment reduces the survival of 5-HT neurons in the rostral raphe and substantia nigra (Jarskog et al., 1997). Exposure to maternal obesity-induced inflammation may increase risk for mental health disorders in offspring by suppressing serotoninergic function.

#### Maternal obesity impairs development of reward neural circuitry

The role of maternal obesity on central reward signaling has only been examined in one study (Stice et al., 2011). There, normal-weight adolescents with two overweight or obese parents had increased neural activation of the caudate putamen and frontal and parietal cortex after consuming a milkshake. Human research demonstrates that maternal obesity is linked to impairments in central reward signaling in adolescents. The role of GWG, maternal metabolic disorders, and an unhealthy maternal diet on central reward signaling remain unknown. Existing animal literature agrees with human literature on maternal obesity impairing central DA signaling in offspring. In rodent studies, research focuses on the mesolimbic and mesocortical DA neural circuit, which includes the ventral tegmentum, nucleus accumbens, and prefrontal cortex. Maternal HFD led to abnormal central DA signaling in offspring. Existing rodent literature is mixed as to the direction of this effect. Two studies point to maternal HFD potentiating central DA signaling. Maternal HFD increased protein levels of the rate-limiting enzyme tyrosine hydroxylase (which increases synthesis of DA) in the ventral tegmentum (Naef et al., 2008) and DA release to acute stress in the nucleus accumbens (Naef et al., 2013a). A second group of studies found maternal HFD suppressed central DA signaling. Maternal HFD decreased DA responses in anticipation to food (Naef et al., 2013b) and expression of tyrosine hydroxylase, dopamine receptor 1 (G protein-coupled receptor that increases DA release), and dopamine receptor 2 (G protein-coupled receptor that decreases DA release) mRNA in the nucleus accumbens (Vucetic et al., 2010). Dopamine receptor 1 and 2 were also decreased in the prefrontal cortex. We hypothesize that increased maternal obesity-induced inflammation suppresses development of the DA neural circuit, as treatment with inflammatory cytokines, interleukin-6, IL-1β, and tumor necrosis factor-α, decrease the survival of DA neurons in the substantia nigra (Jarskog et al., 1997). This inflammation-induced impairment in central DA signaling is presumably what increases the risk for reward-related behaviors in offspring.

#### Maternal obesity impairs development of melanocortin neural circuit

The melanocortin neural circuit in the arcuate nucleus of the hypothalamus and the paraventricular nucleus of the hypothalamus regulates energy intake (Cone, 1999). Alpha-melanocyte stimulating hormone (a cleavage product of the proopiomelanocortin gene) inhibits food intake through activation of melanocortin receptor subtype 3 and melanocortin receptor subtype 4 (MC4R). In contrast, agouti-related peptide (AgRP) potentiates food intake through blockade of melanocortin receptor subtype 3 and MC4R. AgRP is colocalized with neuropeptide Y. No human literature exists examining the impact of maternal obesity on central melanocortin signaling in children. Rodent studies have pointed to maternal HFD impairing central melanocortin signaling in offspring. However, findings are inconsistent as to whether POMC or AgRP is affected and to the direction of this effect. One study found maternal HFD increased expression of AgRP/neuropeptide Y, POMC, and MC4R mRNA in the entire hypothalamus (Gupta et al., 2009), whereas two separate studies found that it decreased the expression of AgRP/neuropeptide Y mRNA in the arcuate nucleus of the hypothalamus and entire hypothalamus (Chang et al., 2008; Sun et al., 2014). Species differences are apparent in the ontogeny of this neural circuit. The melanocortin neural circuit develops as late as the third postnatal week in rodents (Grove et al., 2003; Grove and Smith, 2003), but during the third trimester of pregnancy in primates (NHP and humans), making the melanocortin circuit of primates more vulnerable to changes in the maternal environment (Koutcherov et al., 2003; Grayson et al., 2006). Using a NHP model, our laboratory found that third trimester fetal offspring whose dams consumed a HFD had decreased expression of AgRP mRNA and protein, but increased expression of POMC and MC4R mRNA in the arcuate nucleus of the hypothalamus (Grayson et al., 2010). Inflammatory mechanisms also appear to lead to impairments in central melanocortin signaling. In the rodent, IL-1β, decreases AgRP release and increases POMC release in hypothalamic explants (Scarlett et al., 2007, 2008). This inflammation-induced impairment in central melanocortin signaling is perhaps what increases risk for disordered eating behavior in offspring.

#### A diet high in omega-3 fatty acids improves neural inflammation and function

Diets high in omega-3 fatty acids protect against inflammation in the brain and improve neural function. For instance, omega-3 fatty acid consumption reduced inflammation-induced upregulation of the pro-inflammatory markers (interleukin-6 and tumor necrosis factor-α in microglia (Lu et al., 2010). A decreased inflammatory state in the brain can facilitate improvements in central 5-HT signaling. In particular, consumption of omega-3 fatty acids enhanced 5-HT release from presynaptic neurons and increased cell membrane fluidity in postsynaptic neurons, which may alter serotonin receptor availability in the brain (Patrick and Ames, 2015). Therefore, this evidence demonstrates that a simple dietary intervention may improve central 5-HT signaling and mitigate the risk of developing mental health disorders. Importantly, a reduction in the amount of maternal omega-3 fatty acids is associated with increased risk of ADHD and ASD (Field, 2014), indicating that omega-3 fatty acid supplementation is a promising therapeutic intervention to ameliorate maternal obesity-induced risk of mental health disorders.

## Conclusion

This review highlights research demonstrating that an unhealthy, perinatal environment programs various mental health disorders in offspring with a focus on neurodevelopmental and neuropsychiatric disorders. Epidemiological research demonstrates a link between maternal obesity and the development of mental health disorders, including ADHD, ASD, anxiety, depression, schizophrenia, eating disorders (food addiction, anorexia nervosa, and bulimia nervosa), and cognition. There is no literature demonstrating a link between maternal obesity and substance addiction, indicating a need for further research in this area. There is also evidence that excessive GWG, maternal metabolic disorders, and an unhealthy maternal diet increase the risk for ADHD, ASD, schizophrenia, and eating disorders. However, these metabolic conditions have been poorly characterized in anxiety, depression, and cognition. Animal research agrees with human studies and shows that maternal obesity (produced by HFD consumption) contributes to the development of hyperactivity, decreased sociability, increased anxiety-like and depressive-like behavior, substance addiction, food addiction, and decreased cognition in offspring. Schizophrenia, anorexia nervosa, and bulimia nervosa have yet to be examined in animals. Animal models also provide insight into the mechanisms by which maternal obesity impacts offspring behavior. Maternal obesity has been shown to increase fetal exposure to inflammation resulting in impairments in the development of critical neural circuits that regulate behavior. Impairments in these neural pathways appear to lead to the development of mental health disorders in offspring. Future animal studies need to identify the biological mechanisms involved and whether they differ depending on the risk factor. Lastly, observational studies have been primarily conducted in human research. Future studies should focus on randomized controlled trials, to determine whether early dietary interventions prevent the development of mental health disorders in offspring. Specific dietary interventions, like omega-3 fatty acid supplementation, provide a cost-effective means to reduce the prevalence of mental health disorders in children.

### Conflict of interest statement

The authors declare that the research was conducted in the absence of any commercial or financial relationships that could be construed as a potential conflict of interest.

## Abbreviations

5-HT: serotonin
ADHD: attention deficit hyperactivity disorder
AgRP: agouti-related peptide
ASD: autism spectrum disorder
BMI: body mass index
DA: dopamine
GWG: gestational weight gain
HFD: high-fat diet
IL-1β: interleukin-1β
NHP: nonhuman primate
MC4R: melanocortin receptor subtype 4
POMC: proopiomelanocortin.
